# Age-Stratified Clinicopathological Features and Efficacy of Adjuvant Chemotherapy in Resectable Gastric Cancer: An East-West Population-Based Study

**DOI:** 10.3390/curroncol32090480

**Published:** 2025-08-26

**Authors:** Zijian Deng, Jianping Guo, Zhizhong Xiong, Bin Zhong, Dayin Huang, Haoyang Xu, Shi Chen, Lei Lian

**Affiliations:** 1 Department of General Surgery (Department of Gastrointestinal Surgery), The Sixth Affiliated Hospital, Sun Yat-sen University, Guangzhou 510655, China; 2 Guangdong Provincial Key Laboratory of Colorectal and Pelvic Floor Diseases, The Sixth Affiliated Hospital, Sun Yat-sen University, Guangzhou 510655, China; 3 Biomedical Innovation Center, The Sixth Affiliated Hospital, Sun Yat-sen University, Guangzhou 510655, China; 4 Department of Gastrointestinal Surgery, The Sixth Affiliated Hospital, Sun Yat-sen University Yuexi Hospital, Xinyi 525399, China

**Keywords:** early-onset gastric cancer (EOGC), adjuvant chemotherapy (AC), population-based study, propensity score matching

## Abstract

**Background:** The incidence of early-onset gastric cancer (EOGC) has been steadily increasing in recent years. However, the efficacy of adjuvant chemotherapy (AC) in this population remains unclear. This study aimed to investigate the clinicopathological characteristics, survival outcomes, and efficacy of AC between EOGC and average-onset gastric cancer (AOGC) patients. **Methods:** Patients with stage II–III gastric adenocarcinomas who underwent curative D2 gastrectomy at the Sixth Affiliated Hospital of Sun Yat-sen University from January 2014 to December 2021 were enrolled and classified into two groups: EOGC (≤45 years) and AOGC (>45 years) groups. Clinicopathological characteristics, overall survival (OS), and efficacy of AC were compared between the two groups. Western and East Asian cohorts were included as external validation sets to compare the efficacy of AC between different age groups. **Results:** Compared to AOGC, EOGC patients exhibited a higher proportion of females, poor differentiation, diffuse Lauren type, middle-third GC, perineural invasion (PNI), and receipt of AC. Univariate and multivariate analyses identified that T stage, N stage, PNI, and AC were independent prognostic factors for OS. After balancing the baseline characteristics between patients who received AC and those who did not, the Kaplan–Meier survival curves indicated that AC significantly improved OS across all patients. Further subgroup analysis revealed a survival benefit of AC in AOGC patients, whereas no significant survival difference was observed in the EOGC subgroup. Consistently, external validation in both Western and East Asian cohorts confirmed that AC did not confer a survival advantage in EOGC patients. **Conclusions:** EOGC exhibits aggressive pathological characteristics, and chemotherapy does not consistently improve survival in EOGC patients.

## Simple Summary

In this study, we investigate the clinicopathological characteristics, survival outcomes, and efficacy of adjuvant chemotherapy between patients with early-onset gastric cancer and those with average-onset gastric cancer. Compared to 757 patients with average-onset gastric cancer, 130 patients with early-onset gastric cancer were more likely to be female, had more aggressive tumor features (poorer differentiation, diffuse type, perineural invasion), and received chemotherapy more frequently. Adjuvant chemotherapy is an independent protective factor for gastric cancer patients who have undergone radical surgery. However, subgroup analysis by age shows that the efficacy of adjuvant chemotherapy is limited for early-onset gastric cancer, as it cannot significantly improve the overall survival of patients in the early-onset gastric cancer subgroup. The study suggests that early-onset gastric cancer has distinct characteristics and may respond differently to standard chemotherapy. In the future, new treatment strategies should be developed for early-onset gastric cancer.

## 1. Introduction

Gastric cancer (GC) is a major digestive tract malignancy, ranking fifth in global cancer incidence and mortality, with approximately 660,000 deaths reported in 2022 [1]. Historically, GC has been predominantly viewed as a disease primarily affecting older adults, with a median age at diagnosis of 60 years [2,3]. However, a growing number of younger individuals are being affected by GC, a condition typically diagnosed in those under the age of 45 [4]. In China, the incidence rate of early-onset gastric cancer (EOGC) has climbed by 28% over the past twenty years, while that of average-onset GC (AOGC) has declined by 79% [5]. Globally, EOGC accounts for 4.6–14.8% of all GC cases [6], with the United States reporting an exceptionally high prevalence, where over 30% of new diagnoses are EOGC [7]. Moreover, projections indicate that the global incidence of EOGC is poised to nearly double by 2035 [4]. The increasing incidence of EOGC is related to genetics, the environment, and the lifestyles of young people. Approximately 10% of the cases show family clustering with germline mutations in CDH1 or CTNNA1 genes [8]. There is a negative correlation between body mass index and the age at gastric cancer diagnosis [9]. The high incidence of unhealthy lifestyles and metabolic diseases among young people, as well as the high infection rate of Helicobacter pylori (Hp) [10], may all increase the incidence of EOGC.

These evolving trends in GC epidemiology pose formidable challenges to clinicians in determining optimal treatment strategies to improve patients’ survival outcomes. The distinct clinicopathological and molecular profiles of EOGC compared to AOGC suggest that these tumors may represent separate entities in GC [11]. EOGC patients are more prone to high tumor grade, diffuse histological features, high rates of signet-ring cell differentiation, and peritoneal metastases, all of which correlate with a poorer prognosis [4,12]. Consequently, EOGC is frequently diagnosed at advanced stages with limited resectability, resulting in a median overall survival time as short as 11.7 months [13]. This characteristic divergence may lead to differential responses to therapies, including adjuvant chemotherapy (AC) [14].

AC is a cornerstone of treatment for advanced GC after curative resection [15,16,17]. However, current chemotherapy approaches for EOGC patients often follow protocols designed for the general GC population, overlooking the inherent biological heterogeneity of EOGC [18]. This lack of EOGC-specific treatment guidelines is compounded by a dearth of large-scale, multicenter clinical studies [19]. Furthermore, significant knowledge gaps remain regarding the prognostic implications within this population, particularly concerning their response to AC.

In this study, we investigated disparities in the prognostic value of AC between EOGC and AOGC patients using international multi-center cohorts, aiming to assess whether AC provides clinical benefit for EOGC patients. These findings will provide valuable insights into the clinical strategies for EOGC management.

## 2. Materials and Methods

### 2.1. Patient Enrollment

**Figure 1:**
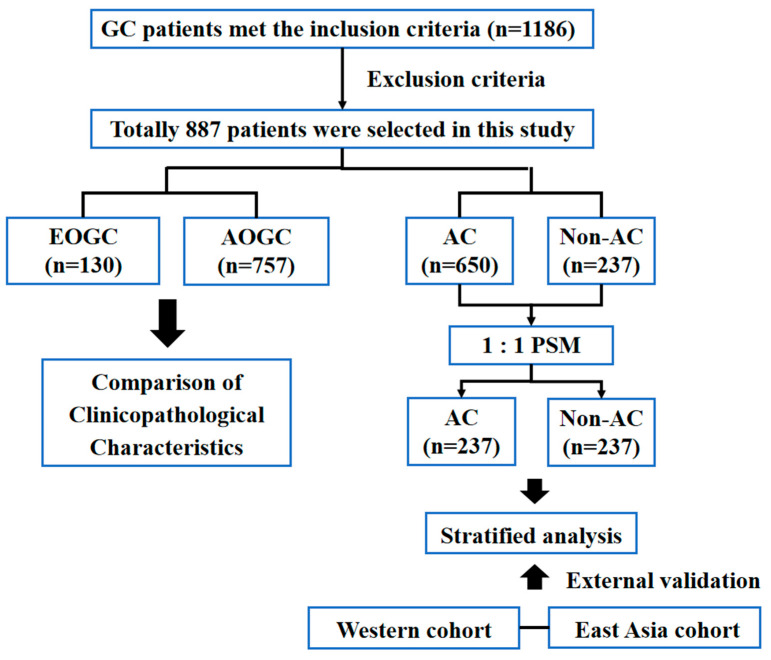
Flowchart of this study.

This retrospective study analyzed GC patients who underwent gastrectomy at the Sixth Affiliated Hospital of Sun Yat-sen University from January 2014 to December 2021. The inclusion criteria were as follows: (1) underwent D2 radical resection with R0 margins; (2) diagnosed with pathological AJCC8th TNM stage II–III gastric adenocarcinoma without evidence of distant metastasis. Exclusion criteria included: (1) severe postoperative complications; (2) prior neoadjuvant chemotherapy, immunotherapy, or radiotherapy before D2 surgery; (3) gastric remnant cancer; (4) lack of essential clinicopathological and follow-up data. A total of 887 patients were enrolled in this study. For external validation, the Surveillance, Epidemiology, and End Results (SEER) database in the United States serves as the Western GC cohort, while the East Asian GC cohorts for external validation included data from the Asian Cancer Research Group (ACRG) [20], Samsung Medical Center (SMC) [21], and Yonsei University [22]. Presented in Figure 1 is a flowchart of the patient enrollment and the process of study implementation.

The study was carried out in accordance with the Helsinki Declaration’s principles. The study received approval from the ethics committee of the Sixth Affiliated Hospital of Sun Yat-sen University.

### 2.2. Clinical Characteristics

Patients diagnosed with gastric adenocarcinoma at age 45 or younger than 45 are classified as EOGC, as defined in previous studies [5,23,24]. Conversely, those diagnosed at an age older than 45 are considered AOGC. All selected GC patients were further subdivided based on their AC status.

Other relevant variables were collected, including sex, tumor differentiation, Lauren type, tumor location, perineural invasion (PNI), lymphovascular invasion (LVI), T stage, N stage, carcinoembryonic antigen (CEA), and carbohydrate antigen 19-9 (CA199).

### 2.3. Propensity Score Matching (PSM)

To summarize the distributions of the variables, descriptive statistics were employed, and categorical variables were compared using either Pearson’s Chi-square test or Fisher’s exact test. Patients who received AC were matched with non-AC patients using PSM. The unbalanced variables, including T stage and N stage, were used as covariates to calculate the individual propensity score. In this study, we employed 1:1 nearest-neighbor matching without replacement, which pairs each non-AC patient with an AC patient having the closest propensity score. The caliper width was set to 0.1.

### 2.4. Statistical Analysis

Overall survival (OS), which refers to the time from surgery until death from any cause or the last follow-up, was the main endpoint of this study. The Survival curves for OS were constructed using the Kaplan–Meier method, and the log-rank test was utilized to assess the differences between these curves.

Univariate Cox regression analysis was used to evaluate the correlations between various factors and the OS in GC patients. To identify independent prognostic factors, variables with potential significance (*p* < 0.05) in the univariate analysis were included in the multivariate Cox proportional hazards regression analysis. Before conducting multivariate Cox regression analysis, we performed the proportional hazards (PH) test on the variables included in the analysis to ensure the validity of the proportional hazards assumption. Statistical significance was determined using a two-tailed *p* value of under 0.05. Data analysis was performed using SPSS version 22.0 and R Project version 4.1.0.

## 3. Results

### 3.1. Patient Clinicopathological Characteristics

The clinicopathological characteristics of EOGC and AOGC patients are summarized and compared in Table 1. Among the 887 enrolled patients, 130 (14.7%) were diagnosed with EOGC, and 757 (85.3%) with AOGC. The proportion of female patients was significantly higher in the EOGC group compared to the AOGC group (47.7% vs. 29.5%, *p* < 0.001). EOGC patients were more frequently associated with poorly differentiated tumors (93.1% vs. 68.0%, *p* < 0.001). Regarding the Lauren classification, EOGC was predominantly of the diffuse type (82.3% vs. 43.1%, *p* < 0.001), whereas AOGC had a higher proportion of intestinal-type (29.2% vs. 4.6%, *p* < 0.001). Additionally, the incidence of PNI was significantly higher in EOGC compared to AOGC (63.8% vs. 52.6%, *p* < 0.001). EOGC was more frequently located in the middle third of the stomach, with fewer cases occurring in the upper stomach (*p* < 0.001). Moreover, AC accounted for 86.9% of the EOGC group, compared with 70.9% in AOGC (*p* < 0.001).

### 3.2. Prognostic Factors in Univariate and Multivariate Analyses

In this study, univariate analysis was conducted across the entirety of GC patients, revealing significant correlations between OS and several variables, including tumor location, T stage, N stage, PNI, LVI, CA19-9 levels, and AC (Table 2). The individual Schoenfeld tests and the Global Schoenfeld Test indicated no evidence of a violation of the PH assumption (Figure S1). Subsequent multivariate Cox proportional hazards regression analyses identified AC as an independent protective factor associated with OS (Hazard ratio [HR] = 0.456, 95% confidence interval [CI]: 0.36–0.58, *p* < 0.001). Additional independent predictors of poorer survival outcomes included T stage, N stage, and PNI.

### 3.3. Stratified Survival Analysis of AC in the Different Age Groups

The results of the above analysis suggested that, despite its association with poorer differentiation, a higher prevalence of diffuse type, and an increased rate of PNI, EOGC did not confer a worse prognosis compared to AOGC (Figure S2). Whether this survival advantage is attributable to a higher proportion of patients receiving chemotherapy remains to be confirmed through further investigation.

To evaluate the effect of AC on patient outcomes across different age groups, we conducted further analyses. Initial comparisons between AC and non-AC groups revealed substantial clinicopathological imbalances, not only in age distribution but also in variables such as tumor differentiation, T stage, N stage, and serum CA19-9 levels. To mitigate potential selection bias, a 1:1 PSM was performed using these imbalanced factors—including T stage and N stage—as covariates. A total of 474 patients were matched. Post-PSM analysis demonstrated that, apart from age, all other clinicopathological variables were well balanced between the AC and non-AC groups (Table 3). Additionally, with the exception of age, all standardized mean differences (SMDs) below 0.2 indicate a good balance between the matched cohorts. For variables of crucial prognostic significance, such as T stage, N stage, and PNI, an even more ideal state of balance (SMD < 0.1) was observed.

**Figure 2:**
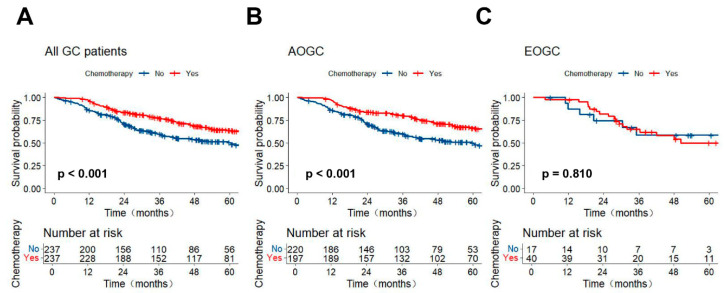
Kaplan–Meier curves for OS of patients from the Sixth Affiliated Hospital of Sun Yat-sen University. (**A**) All patients; (**B**) AOGC patients; (**C**) EOGC patients.

Compared to the non-AC group, the AC group exhibited a significantly better prognosis (Figure 2A, *p* < 0.001). Stratified survival analysis across different age groups further revealed that AOGC patients with AC had a better prognosis compared to those without AC (Figure 2B, *p* < 0.001). However, among EOGC patients, there is no significant survival difference between the AC and non-AC groups (Figure 2C, *p* = 0.810). To further reveal the efficacy of AC for GC patients at different stages, we conducted a subgroup analysis with age as the stratification factor among GC patients at various stages. Similarly, the Kaplan–Meier curve shows that although AC can significantly improve the prognosis of patients with stage II (*p* = 0.005) or stage III (*p* < 0.001) GC who have undergone radical surgery, no significant survival benefit was observed in either stage II (*p* = 0.770) or stage III (*p* = 0.419) GC patients in the EOGC subgroup (Figure S3). In AOGC patients, AC significantly improved OS in both age subgroups (Figure S4): those aged 45–60 years (*p* = 0.018) and those > 60 years (*p* = 0.001).

### 3.4. External Validation of the Efficacy of AC Stratified by Different Age Groups

**Figure 3:**
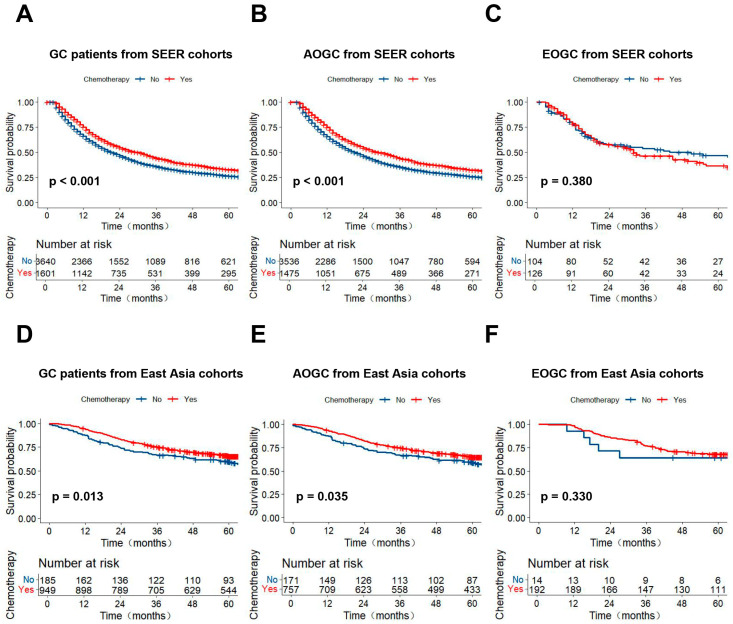
Kaplan–Meier curves for OS of patients in external validation cohorts. (**A**) All patients from the SEER database; (**B**) AOGC patients from the SEER database; (**C**) EOGC patients from the SEER database; (**D**) All patients from East Asia data; (**E**) AOGC patients from East Asia data; (**F**) EOGC patients from East Asia data.

In order to validate these findings, we performed stratified survival analysis using the SEER cohort and East Asia cohorts. As shown in Figure 3, among Western GC patients from the SEER database, AC was associated with survival benefits for the overall GC patients with radical gastrectomy (Figure 3A, *p* < 0.001). However, subgroup analyses indicated that AOGC patients benefited from AC (Figure 3B, *p* < 0.001), while EOGC patients did not (Figure 3C, *p* = 0.380). Similar trends were observed in the East Asia cohorts (Figure 3D–F), where AOGC patients with AC demonstrated significantly prolonged survival compared to the non-AC group (Figure 3E, *p* = 0.035). Conversely, AC failed to extend the OS time of EOGC patients (Figure 3F, *p* = 0.330).

## 4. Discussion

Although global incidence and mortality rates for GC have been declining, there has been a concerning increase in cases among young individuals over the past few decades [25]. Radical gastrectomy followed by AC is the primary treatment approach for resectable GC. Several studies have shown that EOGC tends to be more aggressive and may exhibit chemoresistance [26,27,28]. Nevertheless, there is limited research that stratifies by age to evaluate the response to AC in patients with resectable EOGC. Therefore, we conducted a retrospective study to compare the clinicopathological characteristics and survival outcomes after AC between EOGC and AOGC based on East-West population-based cohorts and found that EOGC and AOGC exhibit distinct clinicopathological features and different responses to AC.

In this study, the clinicopathological characteristics of EOGC aligned with previous reports, including a higher proportion of female patients, a predominance of the diffuse type, poor tumor differentiation, and a high rate of PNI [29,30]. These findings suggest EOGC exhibits more aggressive behavior compared to AOGC. Additionally, EOGC lesions were most frequently located in the middle third of the stomach, consistent with prior observations [26]. Although the underlying mechanism driving the distinct clinicopathological features of EOGC remains unclear, studies have demonstrated the unique role of estrogen in EOGC, potentially contributing to its higher incidence in females [31,32]. Regarding the Hp infection rate, previous studies on EOGC and AOGC have reported inconsistent results [30,33,34]. In the present study, no statistically significant difference in Hp infection rates was found between the two groups (*p* = 0.944, Table S1).

Divergence exists in the survival outcomes of EOGC patients. While some studies have reported a better prognosis for EOGC [35,36], others have found no significant difference between EOGC and AOGC [28,37,38], and some have even suggested that younger patients may experience worse survival outcomes [39,40]. The disparity in prognosis might be attributed to the varying definitions of “early onset” in different studies (e.g., diagnosis under 40, 45, 50, or 60 years old), along with differences in patient inclusion criteria. In this study, we adopted the commonly used age cutoff of 45 years old to define EOGC. Our findings demonstrated that, when compared with AOGC patients, those with EOGC did not show any significant disparities in terms of prognosis. A retrospective study carried out overseas has also revealed that the OS of younger GC patients aged under 45 does not show any statistically significant disparity when compared with that of elderly patients aged over 45 [38]. This conclusion is consistent with the results of our study. Although EOGC is notably more aggressive, its prognosis is equivalent to AOGC patients. The possible reasons were as follows. Younger patients tend to exhibit higher tolerance to both the disease and surgical interventions, whereas older individuals often have poor performance status and a higher susceptibility to postoperative complications, which adversely affect survival outcomes. Another potential reason for the prognostic discrepancy may be related to the tumor stage. EOGC is characterized by higher malignant potential and a significantly higher incidence of metastasis [26]. For stage IV GC patients, the OS time of the EOGC patients was considerably less than that of the elderly patients [30]. Consequently, studies including GC patients across all stages may present a poor prognosis for EOGC due to a high proportion of stage IV cases.

Although EOGC exhibits distinct clinicopathological characteristics, comprehensive treatment strategies specifically tailored for this population have yet to be established. Currently, the treatment regimens for EOGC are essentially the same as those for AOGC. For resectable EOGC patients, radical gastrectomy followed by AC serves as the primary therapeutic strategy. In this study, EOGC patients were more likely to receive AC compared to those with AOGC. In the process of making treatment decisions, both the professional advice of clinicians and the informed consent of patients play a crucial role. Clinicians often recommend AC for EOGC patients due to their relatively better physical status and increased tolerance to the treatment. Moreover, younger individuals tend to be more informed about their illness and chemotherapy options, actively participating in the decision-making process for their treatment. To further investigate whether the superior prognosis of EOGC is attributable to a higher proportion of patients receiving AC, we conducted additional analyses to explore the survival benefits associated with AC in patients of different ages. In the East-West population-based cohorts collectively, GC patients receiving AC demonstrated superior OS compared to those who did not receive AC. However, a subgroup analysis revealed similar survival outcomes between the two groups in EOGC patients. Conversely, among AOGC patients, the AC group showed significantly better prognosis. EOGC is characterized by heightened aggressiveness, often presenting with poorly-differentiated, diffuse-type or signet-ring cell histology. These histological subtypes may inherently exhibit resistance to standard chemotherapy agents. These tumors possess molecular factors that drive chemoresistance, such as CDH1 mutations and mesenchymal phenotypes. In addition, the relatively small sample size of EOGC may affect the statistical power of subgroup analysis. Therefore, we conducted a formal post hoc power analysis for the EOGC subgroup. The results showed that we calculated a statistical power of 0.95. This indicates a 5% probability of failing to detect a clinically significant effect.

This study still has several limitations. First, as a single-center retrospective study, it inevitably introduced selection bias. Second, although we supplemented validation with public East-West population-based datasets, the number of EOGC cases without AC remained limited in stratified analyses. Thus, our findings require further validation through larger-scale cases and prospective studies. Additionally, while all patients received fluorouracil-based adjuvant chemotherapy regimens (XELOX, SOX, FOLFOX, S-1) for at least four cycles, chemotherapy regimens and cycle numbers were heterogeneous across patients. Future studies should standardize both the chemotherapy regimens and the number of cycles administered to clarify their impact on prognosis.

## 5. Conclusions

In conclusion, this multicenter study evaluated the prognostic impact of AC in EOGC and AOGC across Western and East Asian cohorts. Our findings demonstrate that EOGC exhibits more aggressive clinic-pathological features and derives less benefit from AC compared to AOGC. These results underscore the need for distinct therapies tailored to the unique biology of EOGC, offering critical insights for clinical decision-making in this patient population.

## Figures and Tables

**Table 1:** Clinicopathological patient characteristics of EOGC and AOGC.

Characteristic	EOGC *n* = 130 (%)	AOGC *n* = 757 (%)	*p* Value
**Sex**			<0.001
Male	68 (52.3)	534 (70.5)	
Female	62 (47.7)	223 (29.5)	
**Differentiation**			<0.001
Well-Moderate	9 (6.9)	242 (32.0)	
Poor	121 (93.1)	515 (68.0)	
**Lauren Type**			<0.001
Intestinal	6 (4.6)	221 (29.2)	
Mixed	17 (13.1)	210 (27.7)	
Diffuse	107 (82.3)	326 (43.1)	
**Location**			<0.001
Upper	21 (16.1)	241 (31.9)	
Middle	52 (40.0)	175 (23.1)	
Lower	49 (37.7)	325 (42.9)	
Overlap	8 (6.2)	16 (2.1)	
**T stage**			0.382
T1–2	13 (10.0)	82 (10.8)	
T3	83 (63.9)	518 (68.4)	
T4	34 (26.1)	157 (20.7)	
**N stage**			0.318
N0	30 (23.1)	210 (27.7)	
N+	100 (76.9)	547 (72.3)	
**PNI**			0.022
No	47 (36.2)	359 (47.4)	
Yes	83 (63.8)	398 (52.6)	
**LVI**			0.024
No	91 (70.00)	447 (59.0)	
Yes	39 (30.00)	310 (41.0)	
**CEA**			0.131
≤5	120 (92.3)	660 (87.2)	
>5	10 (7.7)	97 (12.8)	
**CA-199**			0.200
≤37	114 (87.7)	626 (82.7)	
>37	16 (12.3)	131 (17.3)	
**Adjuvant chemotherapy**			<0.001
No	17 (13.1)	220 (29.1)	
Yes	113 (86.9)	537 (70.9)	

**Table 2:** Univariate and multivariate Cox regression analyses of overall survival.

Variables	OS			
	Univariable		Multivariable	
	HR (95%CI)	*p* Value	HR (95%CI)	*p* Value
**Age**		0.305		
EOGC	Reference		-	
AOGC	1.187 (0.86–1.65)		-	
**Sex**		0.936		
Male	Reference		-	
Female	1.010 (0.80–1.28)		-	
**Differentiation**				
Well—Moderate	Reference	0.532	-	
Poor	1.040 (0.92–1.18)		-	
**Lauren Type**		0.140		
Intestinal	Reference		-	
Mixed	1.307 (0.95–1.80)		-	
Diffuse	1.308 (0.99–1.73)		-	
**Location**		0.028		0.082
Upper	Reference		Reference	
Middle	1.319 (0.98–1.77)		1.186 (0.88–1.60)	
Lower	1.010 (0.77–1.33)		0.922 (0.70–1.22)	
Overlap	1.907 (1.10–3.30)		1.670 (0.96–2.89)	
**T stage**		<0.001		<0.001
T1–2	Reference		Reference	
T3	2.677 (1.56–4.60)		2.749 (1.57–4.81)	
T4	5.197 (2.96–9.11)		4.328 (2.42–7.75)	
**N stage**		<0.001		<0.001
N0	Reference		Reference	
N+	2.173 (1.63–2.90)		2.664 (1.97–3.61)	
**PNI**		<0.001		0.001
Negative	Reference		Reference	
Positive	1.899 (1.51–2.39)		1.490 (1.18–1.91)	
**LVI**		<0.001		0.242
Negative	Reference		Reference	
Positive	1.511 (1.21–1.89)		1.150 (0.91–1.45)	
**CEA**		0.282		
**≤5**	Reference		-	
**>5**	1.200 (0.86–1.67)		-	
**CA19-9**		0.015		0.411
**≤37**	Reference		Reference	
**>37**	1.408 (1.07–1.86)		1.125 (0.85–1.49)	
**Adjuvant chemotherapy**		0.001		<0.001
No	Reference		Reference	
Yes	0.544 (0.43–0.68)		0.456 (0.36–0.58)	

**Table 3:** Clinicopathological patient characteristics between AC and non-AC GC patients.

Characteristic	Before PSM		*p* Value	SMD	After PSM		*p* Value	SMD
	Non-AC *n* = 237 (%)	AC *n* = 650 (%)			Non-AC *n* = 237 (%)	AC *n* = 237 (%)		
**Age**			<0.001	0.269			<0.001	0.259
≤45	17 (7.2)	113 (17.4)			17 (7.2)	40 (16.9)		
>45	220 (92.8)	537 (82.6)			220 (92.8)	197 (83.1)		
**Sex**			0.667	0.039			0.921	0.009
Male	164 (69.2)	438 (67.4)			164 (69.2)	165 (69.6)		
Female	73 (30.8)	212 (32.6)			73 (30.8)	72 (30.4)		
**Differentiation**			0.048	0.152			0.373	0.083
Well-Moderate	79 (33.3)	172 (26.5)			79 (33.3)	70 (29.5)		
Poor	158 (66.7)	478 (73.5)			158 (66.7)	167 (70.5)		
**Lauren Type**			0.321	0.116			0.325	0.142
Intestinal	69 (29.1)	158 (24.3)			69 (29.1)	55 (23.2)		
Mixed	60 (25.3)	167 (25.7)			60 (25.3)	62 (26.2)		
Diffuse	108 (45.6)	325 (50.0)			108 (45.6)	120 (50.6)		
**Location**			0.267	0.156			0.294	0.171
Upper	62 (26.2)	200 (30.8)			62 (26.2)	81 (34.2)		
Middle	56 (23.6)	171 (26.3)			56 (23.6)	48 (20.3)		
Lower	111 (46.8)	263 (40.5)			111 (46.8)	100 (42.2)		
Overlap	8 (3.4)	16 (2.5)			8 (3.4)	8 (3.4)		
**T stage**			0.027	0.193			1.000	0.001
T1–2	15 (6.3)	80 (12.3)			15 (6.3)	15 (6.3)		
T3	164 (69.2)	437 (67.2)			164 (69.2)	164 (69.2)		
T4	58 (24.5)	133 (20.5)			58 (24.5)	58 (24.5)		
**N stage**			<0.001	0.427			1.000	0.001
N0	95 (40.1)	145 (22.3)			95 (40.1)	95 (40.1)		
N+	142 (59.9)	505 (77.7)			142 (59.9)	142 (59.9)		
**PNI**			0.359	0.075			0.581	0.051
No	115 (48.5)	291 (44.8)			115 (48.5)	109 (46.0)		
Yes	122 (51.5)	359 (55.2)			122 (51.5)	128 (54.0)		
**LVI**			0.294	0.085			0.774	0.026
No	151 (63.7)	387 (59.5)			151 (63.7)	154 (65.0)		
Yes	86 (36.3)	263 (40.5)			86 (36.3)	83 (35.0)		
**CEA**			0.498	0.061			0.487	0.066
≤5	205 (86.5)	575 (88.5)			205 (86.5)	210 (88.6)		
>5	32 (13.5)	75 (11.5)			32 (13.5)	27 (11.4)		
**CA 19-9**			0.022	0.190			0.160	0.136
≤37	186 (78.5)	554 (85.2)			186 (78.5)	198 (83.5)		
>37	51 (21.5)	96 (14.8)			51 (21.5)	39 (16.5)		

## Data Availability

The datasets analyzed in this article are included within the article and Supplementary Materials. Please contact the corresponding author for data requests.

## Supplementary Materials

The following supporting information can be downloaded at: https://www.mdpi.com/article/10.3390/curroncol32090480/s1, Figure S1: Scatter plot of Schoenfeld residuals; Figure S2: Kaplan–Meier curves for OS of different age GC patients; Figure S3: Subgroup Kaplan–Meier curves for OS stratified by age among GC patients at different stages; Figure S4: Kaplan–Meier curves for OS of patients aged 45–60 and aged > 60; Table S1: Comparison of Hp infection rates between EOGC and AOGC.

## Abbreviations

The following abbreviations are used in this manuscript:

EOGC	Early-onset gastric cancer
AC	Adjuvant chemotherapy
AOGC	Average-onset gastric cancer
OS	Overall survival
PNI	perineural invasion
GC	Gastric cancer
Hp	Helicobacter pylori
SEER	Surveillance, Epidemiology, and End Results
ACRG	Asian Cancer Research Group
SMC	Samsung Medical Center
LVI	Lymphovascular invasion
CEA	Carcinoembryonic antigen
CA 19-9	Carbohydrate antigen 19-9
PSM	Propensity score matching
PH	proportional hazard
SMD	Standardized mean differences
