# Supplementary material for: Age-Stratified Clinicopathological Features and Efficacy of Adjuvant Chemotherapy in Resectable Gastric Cancer: An East-West Population-Based Study
Source: Curr Oncol. 2025 Aug 26;32(9):480. doi: 10.3390/curroncol32090480 (PMC12468768; doi:10.3390/curroncol32090480)
Supplement: Supplementary file 1 [file curroncol-32-00480-s001.zip › curroncol-3763830-supplementary.pdf]

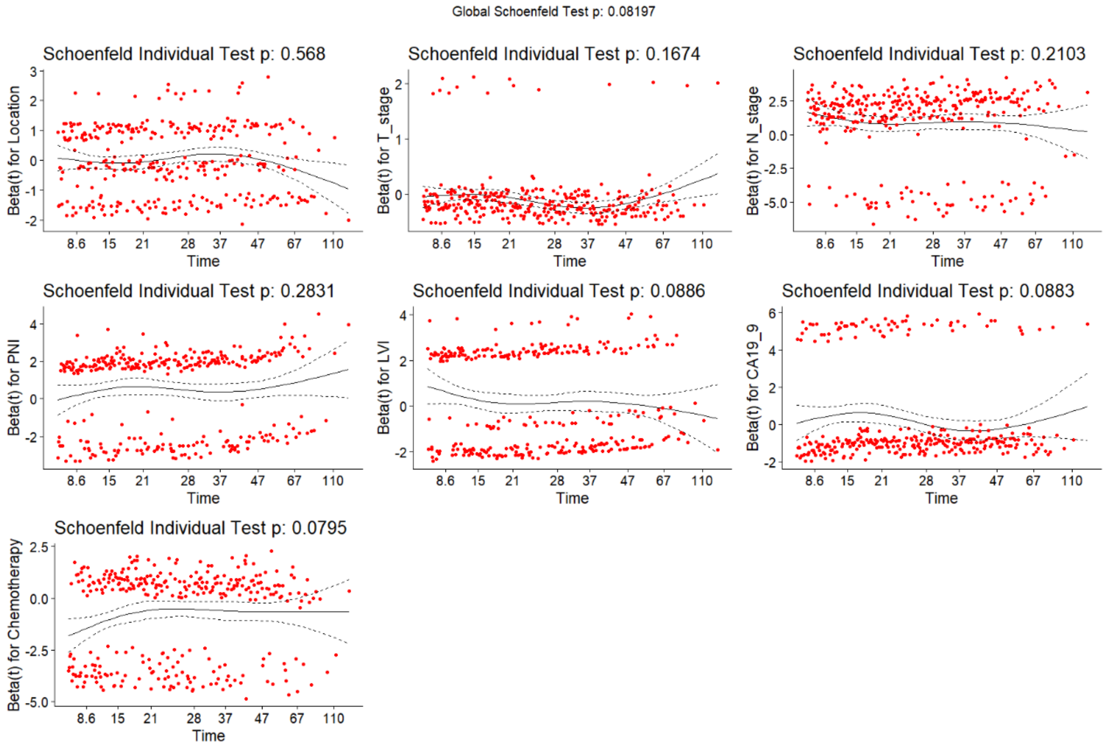

Figure S1. Scatter plot of Schoenfeld residuals

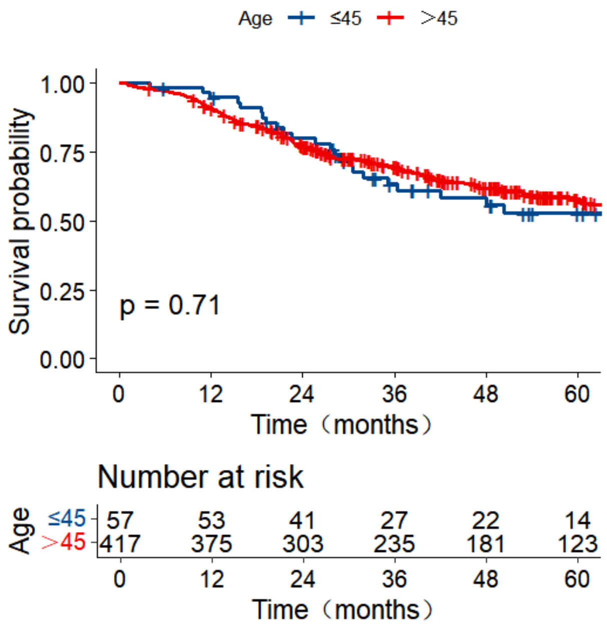

Figure S2. Kaplan-Meier curves for OS of different age GC patients.

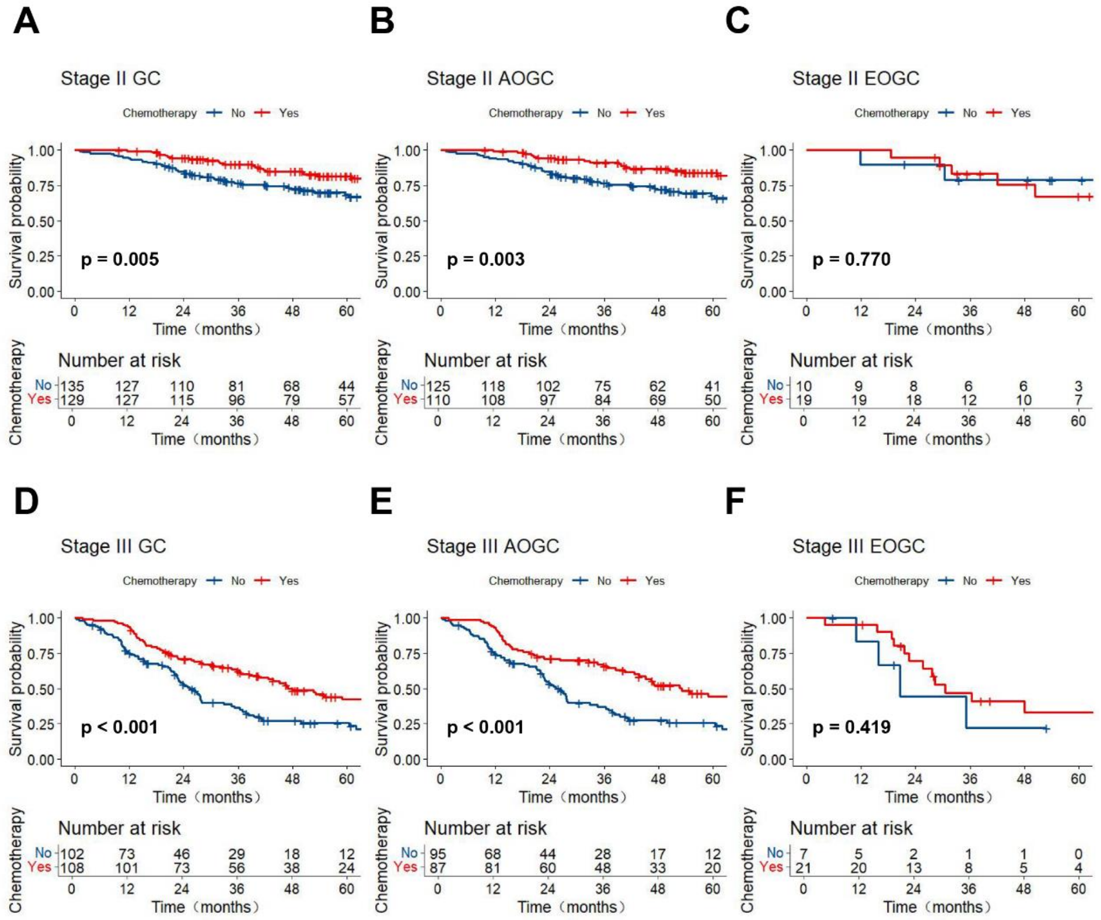

Figure S3. Subgroup Kaplan-Meier curves for OS stratified by age among GC patients at different stages. A) Stage II GC patients; B) AOGC patients in stage II; C) EOGC patients in stage II; D) Stage III GC patients; E) AOGC patients in stage III; F) EOGC patients in stage III.

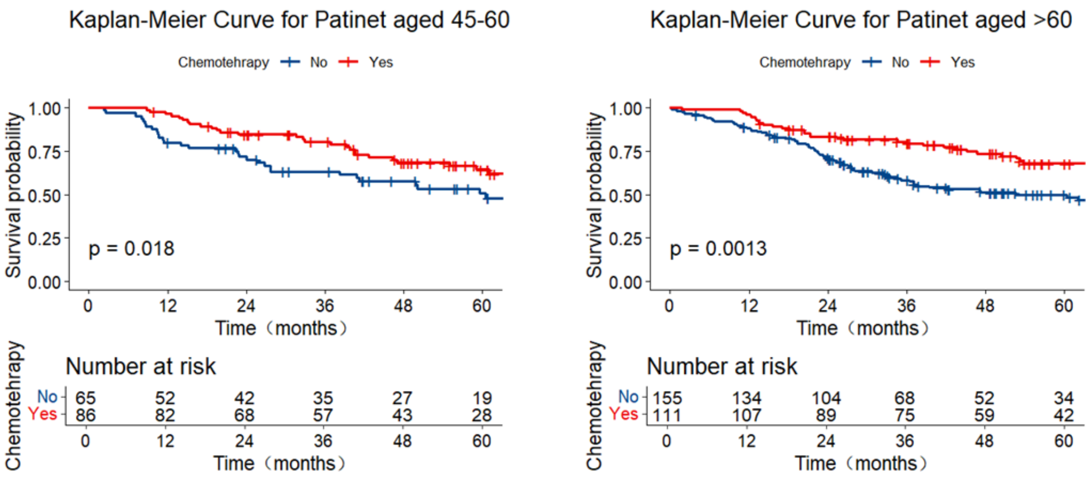

Figure S4. Kaplan-Meier curves for OS of patients aged 45-60 and aged >60.

Table S1. Comparison of Hp infection rates between EOGC and AOGC.

**Characteristic**	**EOGC** n=130 (%)	**AOGC** n=757 (%)	***P* value**
**Hp infection**			0.944
Positive	11 (8.5)	71 (9.4)	
Negative	104 (80.0)	598 (79.0)	
Unknow	15 (11.5)	88 (11.6)	
